# Supplementary material for: Complexome profiling of the Chlamydomonas psb28 mutant reveals TEF5 as an early PSII assembly factor
Source: Plant Cell. 2025 Mar 17;37(6):koaf055. doi: 10.1093/plcell/koaf055 (PMC12164590; doi:10.1093/plcell/koaf055)
Supplement: koaf055_Supplementary_Data [file koaf055_supplementary_data.zip › psb28_interactive.zip!/plots/intensity/qualityPlots/peptides/NUO17_Cre05.g240800.t1.2.html]

### Cre05.g240800.t1.2

Trivial name: NUO17  
SeqCoverage [median]: 51.70  
Mass: 19.60 kDa  
  
 

| Sequence | MissedCleavage | Mass [Da] | wtIntensity | muIntensity | Score |
| --- | --- | --- | --- | --- | --- |
| AAKELETEMR | 1 | 1176.58 | 3241.27 | 3860.26 | 183.03 |
| AIYEELEA | 0 | 936.44 | 3490.62 | 7787.74 | 87.58 |
| DLNMIEDEAYDLVLMR | 0 | 1938.91 | 664.69 | 0.00 | 49.56 |
| ELETEMR | 0 | 906.41 | 4945.16 | 0.00 | 97.07 |
| GGGGEYPGGSFWSEGTQTGK | 0 | 1957.84 | 634.26 | 726.41 | 51.21 |
| LGMHTGRVPEDLK | 1 | 1451.76 | 0.00 | 4905.06 | 61.11 |
| LNERPDLK | 1 | 983.54 | 290.40 | 0.00 | 93.37 |
| MLDKLNERPDLKER | 3 | 1755.93 | 3168.22 | 2227.95 | 72.89 |
| PLEALDIK | 0 | 897.52 | 1042.44 | 0.00 | 77.06 |
|  |  |  | 17477.1 | 19507.4 |  |
